# Spending, utilization, and price trends for anti-obesity medications in U.S. Medicaid programs: an empirical analysis from 1999 to 2023

**DOI:** 10.3389/fmed.2025.1537181

**Published:** 2025-07-16

**Authors:** Abdulrahman A. Alsuhibani, Marwan A. Alrasheed, Ibrahim S. Alhomoud, Saud Alsahali, Ziyad S. Almalki, Jeff Jianfei Guo

**Affiliations:** ^1^Department of Pharmacy Practice, College of Pharmacy, Qassim University, Buraydah, Saudi Arabia; ^2^Department of Clinical Pharmacy, College of Pharmacy, King Saud University, Riyadh, Saudi Arabia; ^3^Department of Clinical Pharmacy, College of Pharmacy, Prince Sattam Bin Abdulaziz University, Al-Kharj, Saudi Arabia; ^4^James L. Winkle College of Pharmacy, University of Cincinnati Academic Health Center, Cincinnati, OH, United States

**Keywords:** obesity, pandemic, utilization, spending, trends, Medicaid, COVID-19

## Abstract

**Background:**

Obesity poses a significant public health and economic challenge in the United States (U.S.), with rising prevalence, particularly among individuals enrolled in Medicaid—the nation’s public health insurance program for low-income populations. Anti-obesity medications (AOMs) have become integral to managing obesity, but trends in their utilization and spending within Medicaid remain underexplored.

**Objective:**

To examine Medicaid’s utilization, reimbursement, and price trends for AOMs from 1999 to 2023.

**Methods:**

A retrospective analysis assessing the utilization, reimbursement, and pricing of older and newer AOMs. Yearly prescription numbers and reimbursement were calculated for seven AOMs billed through Medicaid between 1999 and 2023. The average expenditure per prescription was used as an indicator of drug pricing.

**Results:**

AOM prescriptions rose from 13,855 in 1999 to 938,663 in 2023, a 6,674% increase. Spending surged by over 77,805,466% due to the introduction of newer, more effective medications, including Wegovy and tirzepatide. The largest share of the market growth in 2023 was driven by these medications.

**Conclusion:**

The significant increase in AOM utilization and spending highlights the growing burden of obesity on Medicaid, emphasizing the need for policy measures to manage rising costs and ensure equitable access to treatment.

## Introduction

Obesity, defined as a body mass index (BMI) of ≥30 kg/m^2^, is a multifactorial condition driven by complex genetic, environmental, and neurobiological factors that create an imbalance between energy intake and expenditure. Obesity remains a significant public health issue in the United States (U.S.) and globally (1). Not only does it pose a clinical threat, but it also represents a substantial economic burden on healthcare systems, affecting patients’ quality of life. As of 2023, 41.9% of U.S. adults are classified as obese, marking an increase from prior years (1). This growing prevalence is especially pronounced among minority populations such as African Americans and Hispanics, who have obesity rates of 49.9 and 45.6%, respectively (2). Additionally, obesity rates are notably higher in rural communities compared to urban areas (3). Children and adolescents are also affected, with approximately 20% of individuals aged 2 to 19 living with obesity, a rate that has more than tripled since the mid-1970s (4). The disparities extend across states, with West Virginia (41%), Louisiana (40.1%), and Oklahoma (40%) experiencing the highest adult obesity rates, while states like Colorado (25%) and Hawaii (25.9%) report the lowest using National Health and Nutrition Examination Survey (NHANES) data (5, 6). A notable misconception about weight status exists among individuals with obesity. A survey that included 1,509 participants showed that approximately 89% of those who met the criteria for obesity considered themselves as overweight (7). These trends and misconceptions highlight the ongoing challenges that obesity poses to public health, especially among vulnerable populations.

The global prevalence of obesity has tripled over the past 50 years, driven by factors such as increased access to processed foods, shifts in dietary patterns, and more sedentary lifestyles (8). The obesity pandemic impacts all populations from diverse socioeconomic backgrounds (9–11). For instance, over one-third of the populations in countries like South Africa, the U.S., Mexico, and Saudi Arabia are now living with obesity (12). This underscores the urgent need for effective strategies to mitigate the growing burden of this disease, particularly given its association with an increased risk of cardiovascular disease, hypertension, type 2 diabetes, obstructive sleep apnea, and certain cancers, among other health complications (12).

Nutritional therapies, physical activities, behavioral therapies, and medical interventions (including pharmacotherapy and surgical interventions) are the four pillars of obesity management (13). Despite education and environmental interventions aimed at obesity prevention, maintaining long-term weight loss remains challenging with lifestyle modifications alone (14). Multimodal treatment strategies are essential for patients with obesity to ensure effective obesity management. Therefore, pharmacological management of obesity aims to augment lifestyle modifications (15). Current guidelines recommend anti-obesity medications (AOMs) for individuals with BMI of ≥27 kg/m (2) with comorbidities or BMI ≥ 30 kg/m^2^ (16–18). Bariatric surgery has proven to be an effective intervention for severe obesity, improving survival rates for patients who qualify (19). However, concerns regarding surgical risks and perioperative complications, along with limited insurance coverage and patient hesitancy, pose significant barriers to broader access to this procedure (20–23).

The growing recognition that lifestyle changes alone are often insufficient to address obesity has driven an increased focus on the use of pharmaceutical treatments as a complementary approach. Randomized controlled trials (RCTs) have demonstrated that both oral and injectable AOMs can lead to significant weight reduction; however, the degree of weight reduction from AOMs may not reach the same level as that achieved with bariatric surgery (24). While new drugs, particularly incretin-based therapies, have shown potential in promoting significant weight loss and improving patient quality of life, they also come with a higher price tag (25). Additionally, long-term use of these agents is essential for maintaining weight loss benefits. Discontinuation of therapy for reasons such as cost may lead to weight regain (26). This has a direct impact on the costs linked to obesity treatment, especially within Medicaid, where financial resources are often constrained. With the obesity epidemic continuing to rise globally and clinical data indicating the effectiveness of new AOMs, improving access to these medications is crucial.

The global economic burden of obesity is estimated to be 2 trillion USD each year and approximately 2.8% of the global gross domestic product (GDP) (27). Analysis from the Organization for Economic Co-operation and Development (OECD) countries estimates that 8.4% of healthcare budgets will be spent on managing overweight-related diseases (28). Of these expenditures, 70% will be to cover diabetes treatment, 23% to cardiovascular diseases, and 9% to cancer management (28). Well-established evidence links obesity to diabetes, cardiovascular diseases, and cancers (29). The economic burden of obesity in the United States is the highest globally based on a single-year estimate using the Value of a Statistical Life (VSL) (30). This shows the importance of addressing and managing obesity through a comprehensive management approach, including the use of AOMs. The impact of publicly financed anti-obesity pharmacotherapy is also significant and projected to reach $44.0 billion by 2030 (31). The annual cost of older generation AOMs, such as phentermine-topiramate and orlistat, ranges between $1,500 and $8,500, while newer generation treatments exceed $12,000 USD (25). The cost of these medications varies substantially across countries. For instance, in Japan, a 4-week supply of semaglutide is priced at $283, whereas the same supply costs $1,349 in the USA. As a result, most US health insurance do not provide coverage for AOMs except for selected Medicaid programs (32, 33). The number of anti-obesity medication prescriptions reimbursed by state Medicaid programs increased by over 1,300% between 2011 and 2022, largely due to expanded reimbursement of glucagon-like peptide-1 (GLP-1) receptor agonists (34). Other potential reasons include newly approved indications for patients aged 12 years old and older, as well as for patients with obesity and established cardiovascular diseases (15). However, significant barriers to coverage for AOMs still exist globally. Many countries do not provide coverage for the newer generation of AOMs for weight management, primarily due to concerns over cost-effectiveness and the potential economic burden on healthcare budgets (33). The Medical Expenditure Panel Survey (MEPS) data indicates that effective weight loss interventions may offer cost savings. The magnitude of savings demonstrated in the MEPS data was influenced by the percentage of BMI reduction and the baseline BMI. For example, reducing BMI by 15% from a baseline of 35 can generate $1,030 USD annual savings (35).

The rising costs of medications are a significant concern for healthcare systems, particularly in ensuring that essential treatments remain affordable and accessible. Medicaid, a primary healthcare program for low-income individuals, faces increasing challenges as drug costs, including those for anti-obesity treatments, continue to rise (36). An outcome of these rising costs is the low adoption of pharmacotherapy for treating obesity among eligible patients compared to other chronic diseases such as diabetes (37). The expansion of Medicaid under the Affordable Care Act has extended coverage to millions of low-income adults under 65, a demographic particularly affected by obesity (38). The percentage of Medicare fee-for-service (FFS) beneficiaries diagnosed with obesity increased from 6.2% in 2010 to 21% in 2019 (39). Understanding the factors behind these rising costs is crucial for maintaining sustainable budgets while improving access to necessary treatments.

In the USA, Medicaid, the main public financing program for low-income Americans’ healthcare services, experienced a significant growth in membership from 32.2 million to 68.9 million between 1991 and 2015. This makes up about 20% of the U.S. population overall. In terms of demographics, White Americans comprise around 41% of Medicaid beneficiaries, while African Americans make up about 22%. Additionally, women make up about 58% of enrollees, while those who are 17 years of age or younger make up about 48% of the Medicaid-eligible population. These demographic proportions within the total Medicaid enrollment have stayed mostly consistent, despite the fact that the number of Medicaid participants has increased significantly over the previous few decades (40–42). Medicaid is thus comparable in purpose to national health systems or public insurance programs in many other countries, though its benefits and coverage vary by U.S. state.

Despite the growing demand for effective obesity treatments, research on trends in the utilization, spending, and pricing of anti-obesity medications remains scarce. No studies have specifically analyzed these trends within Medicaid, leaving the scope of usage and reimbursement unclear. Many analyses have failed to account for both older and newer AOMs, creating a gap in understanding the full landscape of obesity treatment in real-world settings. As the prevalence of obesity continues to increase, so does the financial burden associated with its treatment. With longer treatment durations and rising medication costs, particularly in resource-constrained settings like Medicaid, addressing these gaps becomes even more crucial. This study aims to investigate trends in the utilization, expenditure, and pricing of anti-obesity medications from April 1999 to December 2023, providing critical insights that could inform policies aimed at optimizing healthcare resource allocation, enhancing patient care, and mitigating the growing economic burden on healthcare systems.

## Methods

The study employed a descriptive, retrospective drug utilization analysis covering the period from the second quarter of 1999 through the end of 2023. Data for this study were collected from the Medicaid State Drug Utilization Data provided by the Centers for Medicare & Medicaid Services (CMS). This comprehensive national database includes quarterly and annual prescription utilization data from all 50 U.S. states and the District of Columbia (43). Each data entry includes an 11-digit National Drug Code (NDC), drug name (brand and generic), the number of outpatient prescription claims, unit doses dispensed, and total pharmacy reimbursement (including drug cost and dispensing fees). The number of days supplied per prescription was not included. All currently approved anti-obesity medications (AOMs) were identified using their brand names and NDC codes. For orlistat, we included the generic version due to its exclusive indication for obesity. For tirzepatide, we used its generic name, as its branded obesity indication (Zepbound) was approved only in late 2023, with only one quarter of data available. It’s worth noting that the initial five digits of the NDC provided insight into the drug manufacturer, while the subsequent digits delineated specific drug products, detailing their strength, dose formulation, and packaging. This observational study was conducted and reported in accordance with the STROBE (Strengthening the Reporting of Observational Studies in Epidemiology) guidelines. Medications selected for analysis had to meet specific criteria. First, only medications that were formally approved by the FDA between 1999 and 2023 to treat obesity were included. Furthermore, the Medicaid database has to have the relevant reimbursement information for these drugs. This study did not include any medications that were prescribed off-label for the treatment of obesity but were not specifically approved by the FDA for such use. The start year, 1999, was chosen because it coincides with the FDA approval of orlistat (Xenical), the earliest AOM widely available and reimbursed by Medicaid. The end year, 2023, represents the most recent available complete year of CMS data at the time of analysis. This extensive 24-year period allowed assessment of long-term utilization trends and captured the introduction and impact of newer-generation anti-obesity medications. Table 1 provides an overview of the U.S. Food and Drug Administration (FDA) approved anti-obesity medications (44, 45).

**Table 1:** Overview of the U.S. FDA-approved anti-obesity medications, 1999–2023.

Generic name	Brand name	FDA approval date	Patent expiration	Manufacturer
Orlistat	Xenical®, and available without prescription a lower dose as Alli®	Xenical, April 1999 Alli, February 2007	Xenical June 2009 Alli NA	Xenical, Cheplapharm Arzneimittel Alli, GlaxoSmithKline LLC
Phentermine/Topiramate	Qsymia®	July 2012	June 2029	Vivus, INC
Naltrexone/Bupropion	Contrave®	September 2014	February 2029	Orexigen Therapeutics INC
Liraglutide	Saxenda®	December 2014	June 2024	Novo Nordisk INC
Setmelanotide	Imcivree®	November 2020	October 2027	Rhythm Pharmaceuticals INC
Semaglutide	Wegovy ®	June 2021	January 2027	Novo Nordisk INC
Tirzepatide	Mounjaro®, Zepbound®	Mounjaro, May 2022 Zepbound, November 2023	Between 2033 and 2039	Eli Lilly

^*^The table outlines the drug category, as well as the generic and brand names, approval dates, patent expiration dates, and manufacturers of individual AOMs. The approval and patent expiration dates are based on U.S. FDA data and U.S. patents, which may differ from those in other countries. Brand names that are not present in the Medicaid databases have been excluded from the table.

Data were extracted from the publicly available Medicaid State Drug Utilization files, which do not contain individual-level patient data. No sampling or exclusion criteria were applied; all available outpatient prescription records for FDA-approved anti-obesity medications (AOMs) from 1999 to 2023 were included in the analysis. Descriptive statistics (means, totals, proportions) were used to summarize trends. Utilization was defined as the total number of outpatient prescriptions dispensed per quarter. Reimbursement referred to the total amount paid by Medicaid for each drug, representing actual program spending on AOMs. Price per prescription was calculated by dividing the total reimbursement by the number of prescriptions, serving as a proxy for the average cost per fill. All financial figures were reported in nominal U.S. dollars (unadjusted for inflation). Joinpoint regression analysis was used to identify statistically significant changes in trends over time.

Trend visualization was conducted using line charts to illustrate changes over the study period. To identify points at which statistically significant shifts occurred, Joinpoint Trend Analysis Software (Version 5.0) was employed. Joinpoint regression was utilized primarily as an exploratory analytic tool to visually and statistically identify key trend inflection points associated with new medication introductions, rather than for detailed cross-drug trend quantification. Annual Percent Change (APC) and Average Annual Percent Change (AAPC) were calculated for each segment. The empirical quantile method was used to address potential non-normality and outliers, with statistical significance determined at a threshold of *p* < 0.05. All analyses were conducted using SAS (Version 9.4) and Microsoft Excel 2019.

## Results

The analysis revealed a significant upward trend in Medicaid utilization and expenditure of anti-obesity medications between 1999 and 2023. The total number of prescriptions for anti-obesity medications increased from 13,855 in 1999 to 938,663 in 2023, marking a 6674.9% rise. Similarly, Medicaid spending on AOMs increased during the study period from $1,473,375.37 million to $1,146,368,046.11 billion, a substantial 77,805,466% increase. The highest annual rise in spending occurred between 2021 and 2022 when expenditures increased significantly from $28,611,599.05 million to $212,287,750.45 million. The introduction of Contrave and Saxenda in 2014 led to a significant increase in the utilization and reimbursement of AOMs, with a 48 and 43% rise, respectively. Additionally, after their launch, the average prescription price increased by 153%. When setmelanotide (Imcivree) was introduced in 2020, it significantly raised the average prescription cost, which had a significant effect on prescription pricing. The most pronounced growth in prescription volume occurred in 2022, following the market entry of Wegovy, resulting in a 536% rise in the number of prescriptions and a 642% increase in reimbursement. This upward trend persisted into the following year with the introduction of tirzepatide, which was associated with a 412% increase in prescription volume and a 440% rise in reimbursement compared to the preceding year (Table 2 summarizes annual data for overall utilization, spending, and prices of anti-obesity medications reimbursed by Medicaid from 1999 to 2023).

**Table 2:** Annual summary data for overall utilization (number of prescriptions), spending, and prices of anti-obesity medications reimbursed by Medicaid, 1999–2023.

Year	Number of prescriptions		Reimbursement		Reimbursement per prescription	
	Utilization (*n*)	Change (%)	Spending ($)	Change (%)	Average price ($)	Change (%)
1999	13,855	--	1473375.37	--	106.3	--
2000	69,301	400.2	7436339.19	404.7	107.3	0.9
2001	82,636	19.2	8812999.65	18.5	106.6	−0.6
2002	69,624	−15.7	7444319.80	−15.5	106.9	0.3
2003	66,925	−3.9	7216938.51	−3.1	107.8	0.9
2004	57,560	−14.0	7507969.37	4.0	130.4	21.0
2005	28,296	−50.8	4305061.22	−42.7	152.1	16.6
2006	21,102	−25.4	3900970.88	−9.4	184.9	21.5
2007	16,947	−19.7	4423268.58	13.4	261.0	41.2
2008	14,430	−14.9	3602349.16	−18.6	249.6	−4.4
2009	11,796	−18.3	3320857.05	−7.8	281.5	12.8
2010	8,563	−27.4	2865764.38	−13.7	334.7	18.9
2011	6,809	−20.5	2843873.23	−0.8	417.7	24.8
2012	6,147	−9.7	2398447.72	−15.7	520.0	24.5
2013	3,854	−37.3	1554471.53	−35.2	545.9	5.0
2014	4,202	9.0	1538099.31	−1.1	731.7	34.0
2015	6,231	48.3	2208482.27	43.6	1851.9	153.1
2016	8,438	35.4	4145357.67	87.7	2001.2	8.1
2017	11,678	38.4	7064146.41	70.4	2033.3	1.6
2018	13,917	19.2	9046120.12	28.1	1983.4	−2.5
2019	14,279	2.6	11071593.21	22.4	2104.5	6.1
2020	18,638	30.5	16132822.26	45.7	2202.7	4.7
2021	28,789	54.5	28611599.05	77.4	28553.0	1196.3
2022	183,064	535.9	212287750.45	642.0	30435.7	6.6
2023	938,663	412.8	1146368046.11	440.0	31207.8	2.5

^*^Utilization reflects the total number of prescriptions dispensed; Reimbursement reflects total Medicaid spending (in nominal USD); Price per prescription is calculated as total reimbursement divided by number of prescriptions.

**Figure 1:**
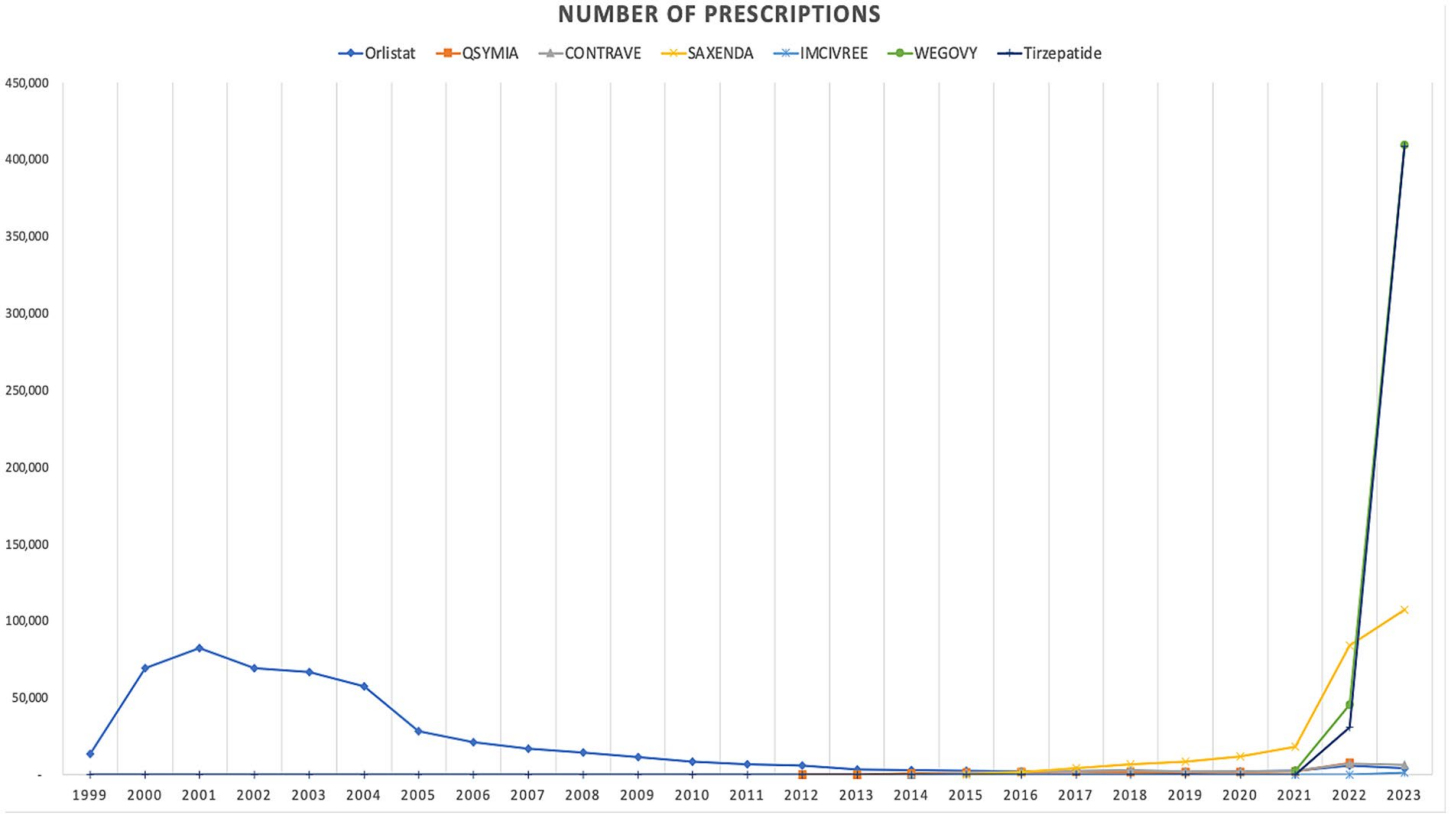
Medicaid utilization (number of prescriptions) of anti-obesity medications from 1999 to 2023.

**Figure 2:**
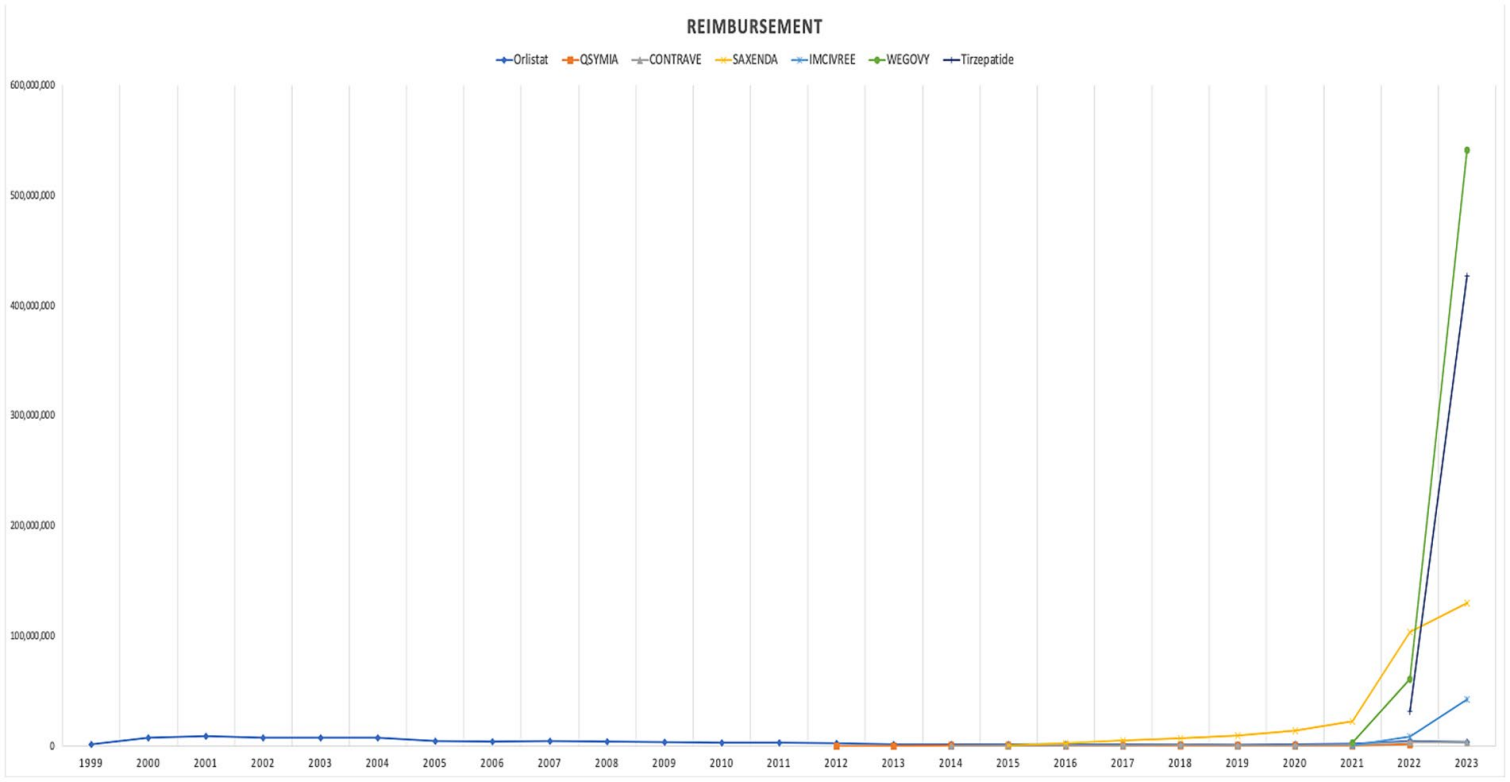
Medicaid spending (reimbursement) of anti-obesity medications from 1999 to 2023 (US$).

In terms of trends, both the utilization and spending on anti-obesity medications experienced a dramatic rise after 2021. The introduction of Wegovy, and tirzepatide medications in in 2021 and 2022 leads this raising trends. Figures 1, 2 illustrate the utilization and spending trends of anti-obesity medications in Medicaid from 1999 to 2023, and Supplementary Figures S1, S2 illustrate the utilization and spending trends of the AOMs in Medicaid, focusing specifically on the years 2013 to 2023.

When comparing individual drugs, orlistat, Wegovy, and tirzepatide dominated the market share utilization, whereas Wegovy, tirzepatide, and Saxenda dominated spending, respectively. Orlistat maintained market dominance until 2013, with a 98% share, but its utilization steadily declined after the introduction of the newer medications (Qsymia, Contrave, and Saxenda). By 2019, orlistat’s market share had dropped to just 8%. The number of prescriptions for orlistat increased from 13,855 in 1999 to 82,636 in 2001, which was its peak. Meanwhile, Wegovy utilization grew from 2,634 to 409,729 prescriptions between 2021 and 2023. The highest growth in prescriptions was observed between 2022 and 2023 for tirzepatide, with prescriptions increasing by 1,214%, from 31,115 to 408,908.

**Figure 3:**
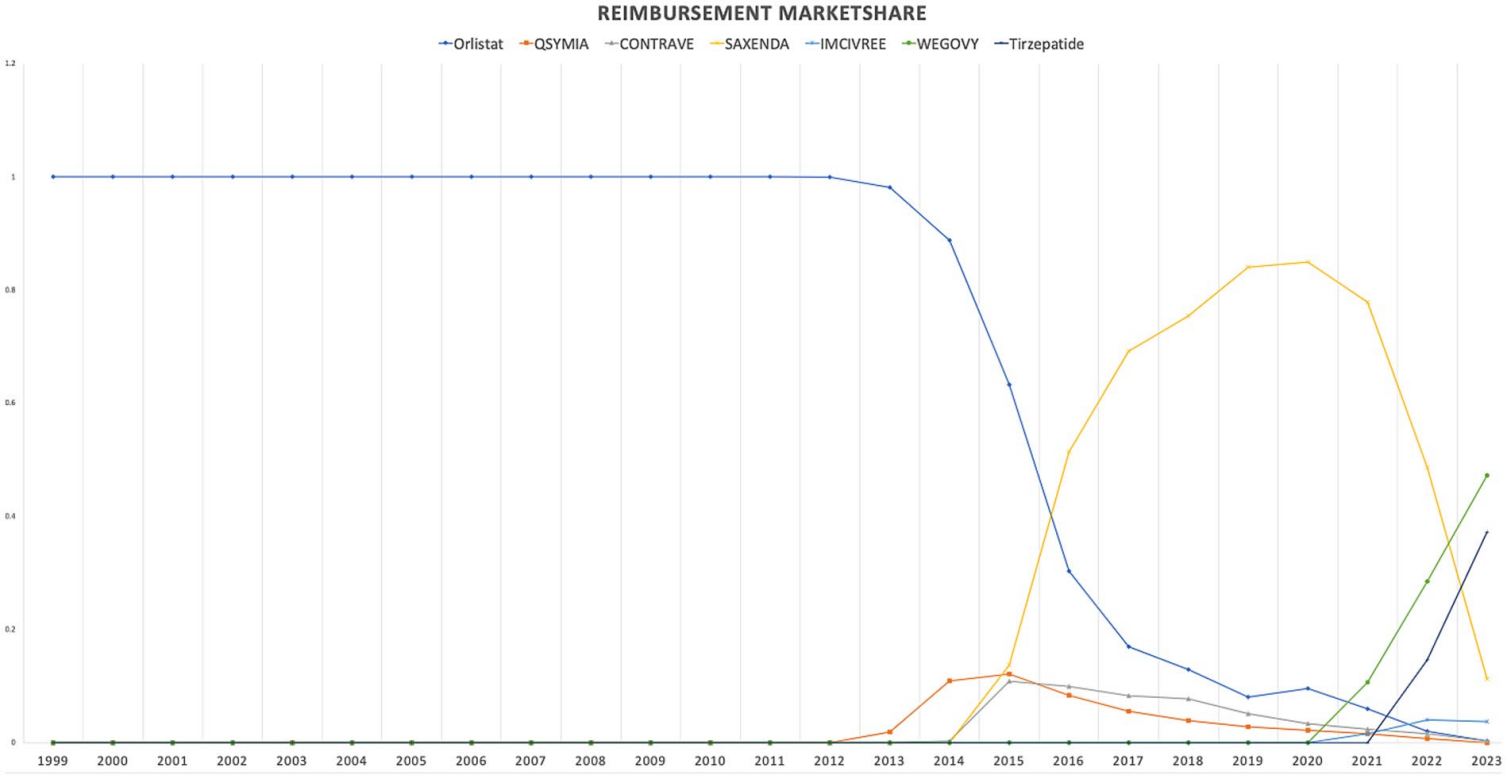
Medicaid utilization (number of prescriptions) market share for anti-obesity medications from 1999 to 2023.

**Figure 4:**
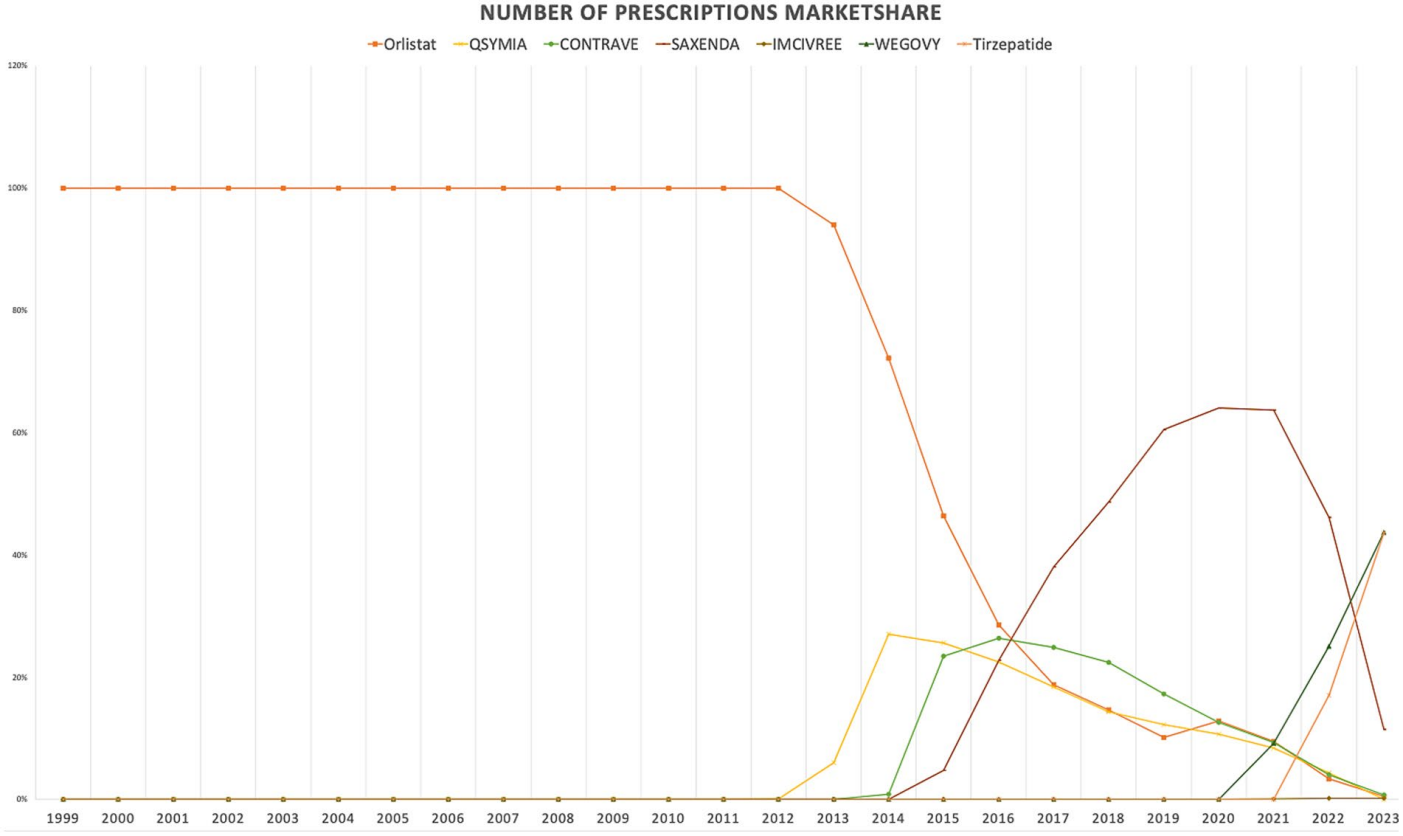
Medicaid spending (reimbursement) market share for anti-obesity medications from 1999 to 2023 (US$).

Qsymia and Contrave saw a peak in utilization in 2015, alongside Saxenda. While the utilization of Qsymia and Contrave declined afterward, Saxenda continued to dominate the market until its peak in 2020, accounting for 64% of the market share. Additionally, Wegovy and tirzepatide accounted for 88% of total prescriptions in 2023, followed by Saxenda at 11%. This reflects the significant market penetration of Wegovy and tirzepatide within just two and one year(s) of entering the market, respectively. In terms of spending market share, Wegovy represented 47%, while tirzepatide accounted for 37% in 2023. Table 3 and Figures 3, 4 summarize the market shares of utilization and reimbursement across the various medications.

**Table 3:** Spending and utilization market shares of individual anti-obesity medications over time, 1999–2023.

Year	Orlistat	Utilization (%)	QSYMIA	Utilization (%)	CONTRAVE	Utilization (%)	SAXENDA	Utilization (%)	IMCIVREE	Utilization (%)	WEGOVY	Utilization (%)	Tirzepatide	Utilization (%)
	Spending (%)		Spending (%)		Spending (%)		Spending (%)		Spending (%)		Spending (%)		Spending (%)	
1999	100%	100%	0%	0%	0%	0%	0%	0%	0%	0%	0%	0%	0%	0%
2000	100%	100%	0%	0%	0%	0%	0%	0%	0%	0%	0%	0%	0%	0%
2001	100%	100%	0%	0%	0%	0%	0%	0%	0%	0%	0%	0%	0%	0%
2002	100%	100%	0%	0%	0%	0%	0%	0%	0%	0%	0%	0%	0%	0%
2003	100%	100%	0%	0%	0%	0%	0%	0%	0%	0%	0%	0%	0%	0%
2004	100%	100%	0%	0%	0%	0%	0%	0%	0%	0%	0%	0%	0%	0%
2005	100%	100%	0%	0%	0%	0%	0%	0%	0%	0%	0%	0%	0%	0%
2006	100%	100%	0%	0%	0%	0%	0%	0%	0%	0%	0%	0%	0%	0%
2007	100%	100%	0%	0%	0%	0%	0%	0%	0%	0%	0%	0%	0%	0%
2008	100%	100%	0%	0%	0%	0%	0%	0%	0%	0%	0%	0%	0%	0%
2009	100%	100%	0%	0%	0%	0%	0%	0%	0%	0%	0%	0%	0%	0%
2010	100%	100%	0%	0%	0%	0%	0%	0%	0%	0%	0%	0%	0%	0%
2011	100%	100%	0%	0%	0%	0%	0%	0%	0%	0%	0%	0%	0%	0%
2012	100%	100%	0%	0%	0%	0%	0%	0%	0%	0%	0%	0%	0%	0%
2013	98%	94%	2%	6%	0%	0%	0%	0%	0%	0%	0%	0%	0%	0%
2014	89%	72%	11%	27%	0.3%	1%	0%	0%	0%	0%	0%	0%	0%	0%
2015	63%	46%	12%	26%	11%	23%	14%	5%	0%	0%	0%	0%	0%	0%
2016	30%	28%	8%	22%	10%	26%	51%	23%	0%	0%	0%	0%	0%	0%
2017	17%	19%	6%	18%	8%	25%	69%	38%	0%	0%	0%	0%	0%	0%
2018	13%	15%	4%	14%	8%	22%	75%	49%	0%	0%	0%	0%	0%	0%
2019	8%	10%	3%	12%	5%	17%	84%	60%	0%	0%	0%	0%	0%	0%
2020	10%	13%	2%	11%	3%	13%	85%	64%	0%	0%	0%	0%	0%	0%
2021	6%	9%	2%	8%	2%	9%	78%	64%	2%	0.1%	11%	9%	0%	0%
2022	2%	3%	1%	4%	2%	4%	49%	46%	4%	0.2%	28%	25%	15%	17%
2023	0.3%	0.5%	0.0%	0.0%	0.3%	1%	11%	11%	4%	0.2%	47%	44%	37%	44%

Regarding the cost per prescription, Imcivree saw the highest average price, at $25,703.6 per prescription. While Wegovy and tirzepatide prescription prices increased by less than 4% between 2022 and 2023. On the other hand, Qsymia had a relatively lower cost during the study period, ranging from $124.8 to $191.6, though data for the year 2023 was unavailable from the source. The peak reimbursement amount per prescription for Saxenda was observed in 2022, while those for orlistat, Contrave, Imcivree, Wegovy, and tirzepatide were recorded in 2023 (Table 4 summarizes average prescription price trends for the included medications).

**Table 4:** Drug prices of individual anti-obesity medications over time, 1999–2023 (reimbursement in dollar amounts per prescription acts as a proxy for average drug price).

Year	Average drug price ($)						
	Orlistat	QSYMIA	CONTRAVE	SAXENDA	IMCIVREE	WEGOVY	Tirzepatide
1999	106.3	--	--	--	--	--	--
2000	107.3	--	--	--	--	--	--
2001	106.6	--	--	--	--	--	--
2002	106.9	--	--	--	--	--	--
2003	107.8	--	--	--	--	--	--
2004	130.4	--	--	--	--	--	--
2005	152.1	--	--	--	--	--	--
2006	184.9	--	--	--	--	--	--
2007	261.0	--	--	--	--	--	--
2008	249.6	--	--	--	--	--	--
2009	281.5	--	--	--	--	--	--
2010	334.7	--	--	--	--	--	--
2011	417.7	--	--	--	--	--	--
2012	390.3	129.7	--	--	--	--	--
2013	421.1	124.8	--	--	--	--	--
2014	450.2	148.2	133.3	--	--	--	--
2015	483.9	168.6	164.1	1035.3	--	--	--
2016	522.8	182.7	185.8	1109.8	--	--	--
2017	548.5	182.1	201.2	1101.5	--	--	--
2018	573.9	176.6	225.1	1007.9	--	--	--
2019	620.6	177.4	228.9	1077.5	--	--	--
2020	646.8	177.8	229.9	1148.2	--	--	--
2021	624.3	186.5	252.3	1215.2	25112.7	1162.0	--
2022	682.9	191.6	452.9	1223.1	25567.7	1320.2	997.2
2023	696.8	--	507.6	1208.5	26430.6	1321.2	1043.2
Average	368.4	167.8	258.1	1125.2	25703.6	1267.8	1020.2

**Figure 5:**
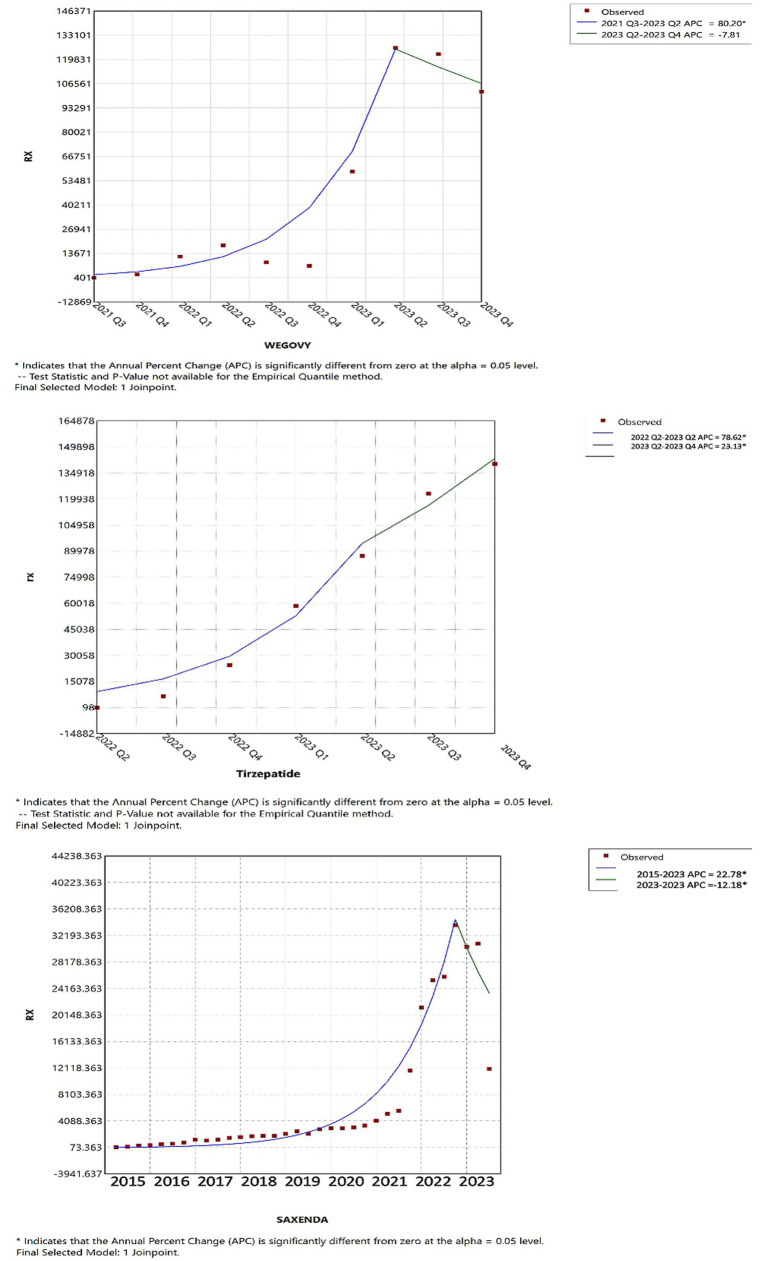
Joinpoint regression for the tirzepatide, Wegovy, and Saxenda AOMs utilization in CMS.

Joinpoint regression analysis revealed notable shifts in the utilization and pricing of anti-obesity medications over time. As illustrated in Figure 5, up until the first quarter of 2023, tirzepatide, Wegovy, and Saxenda demonstrated increasing trends in both utilization and Medicaid reimbursement. However, in the second quarter of 2023, tirzepatide continued to rise significantly in utilization, while Wegovy experienced a modest decline and Saxenda saw a pronounced decrease, suggesting a shift in prescribing patterns.

Joinpoint analysis visually confirmed upward inflection points corresponding to major drug introductions, particularly in 2014 (Contrave and Saxenda), 2021 (Wegovy), and 2022 (tirzepatide). Given the substantial differences among these medications in terms of pricing, market entry timing, and usage trends, detailed year-to-year quantitative comparisons (e.g., APC or AAPC) across products were not emphasized. Such comparisons may be misleading in this context and were beyond the descriptive intent of this study.

**Figure 6:**
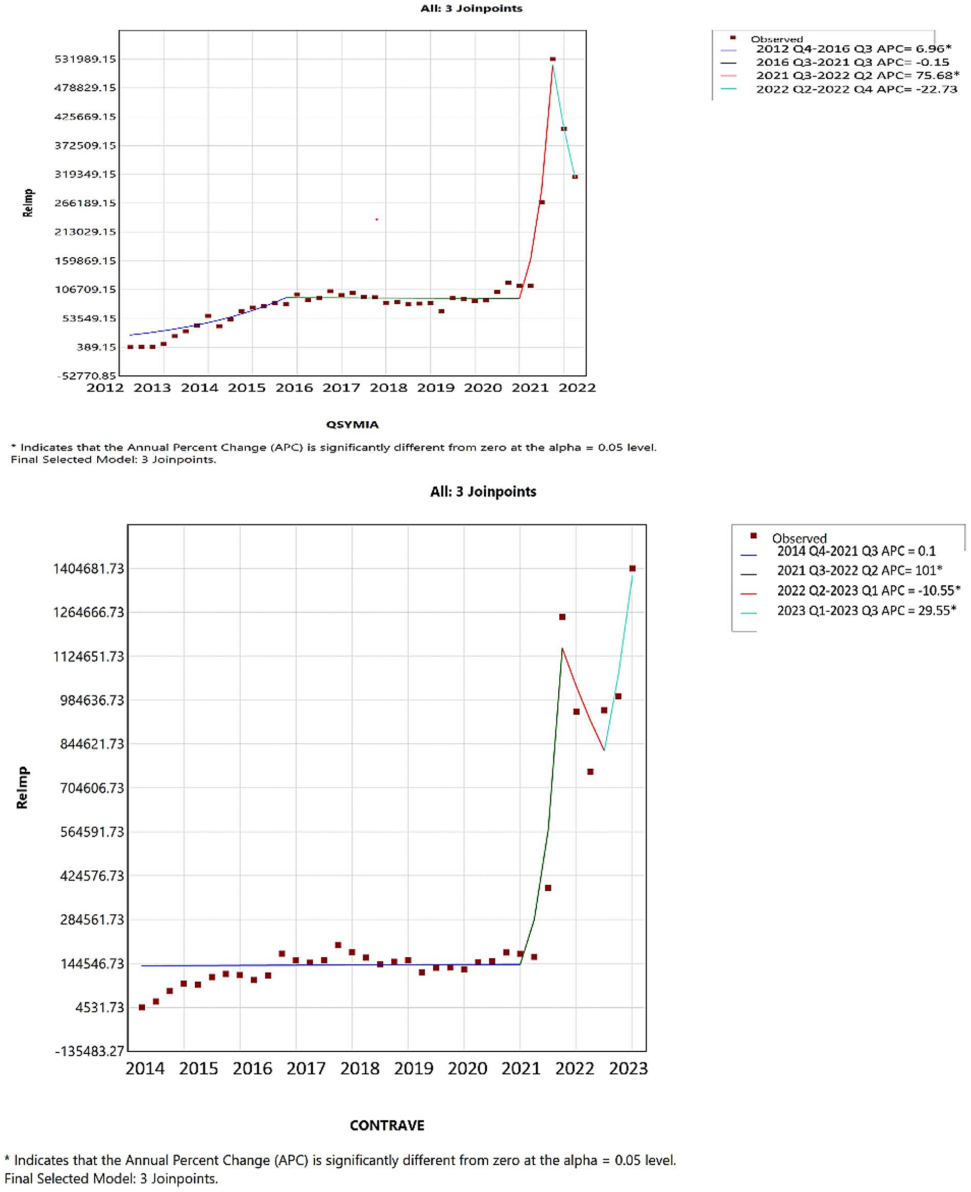
Joinpoint regression for the Qsymia, and Contrave AOMs reimbursement in CMS.

**Figure 7:**
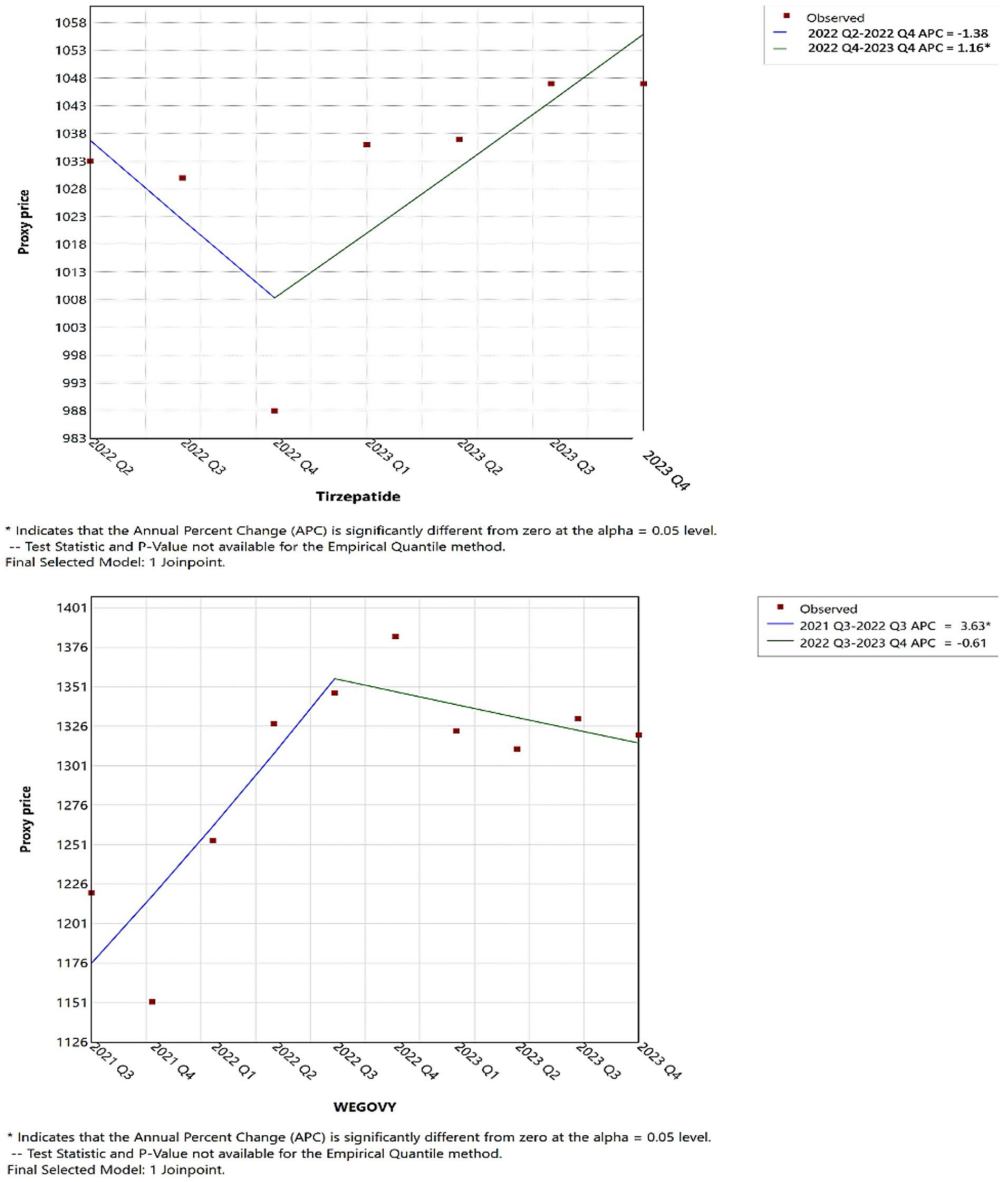
Joinpoint regression for the tirzepatide, and Wegovy AOMs price in CMS.

In 2021s reimbursements, for tirzepatide, Wegovy, and Saxenda mirrored their usage trends; however, Qsymia and Contrave saw a significant reimbursement increase compared to previous periods (Figure 6). Furthermore, as shown in Figure 7, by 2022 Q4, tirzepatide’s prescription price significantly increased, contrasting with a marked price drop for Wegovy during the same period. These results highlight key turning points in both the utilization and pricing of anti-obesity drugs, offering critical insights into market dynamics (further details are available in Supplementary material).

In conclusion, the study demonstrates the growing trend of anti-obesity medication utilization within the Medicaid system, with a significant rise in both the number of prescriptions and overall spending. Future research could explore the underlying drivers of observed trends, including the role of newly introduced medications, reimbursement policies, and clinical guidelines. Additionally, investigations into patient adherence and state-level variation in AOM coverage may offer valuable insights into access disparities and the long-term effectiveness of these treatments in real-world Medicaid populations.

## Discussion

This study is the first to examine utilization, spending, and price trends of anti-obesity medications within the Medicaid program. Our analysis indicates a substantial increase in both spending and utilization of these drugs, the growth was driven primarily by the introduction and subsequent success of Qsymia, Contrave, and Saxenda, followed by the strong impact of Wegovy and tirzepatide. These medications have proven effective in treating obesity and have become integral to treatment protocols in the U.S. healthcare system, particularly among Medicaid patients. The currently approved anti-obesity medications are generally well-tolerated, making them suitable for long-term use in managing obesity.

Our data also highlight the impact of external factors on drug utilization patterns, notably the COVID-19 pandemic. The combination of the pandemic, which underscored obesity as a significant risk factor for worse COVID-19 outcomes, along with the proven efficacy and tolerability of newly approved medications, positively influenced the number of patients who decided and initiated obesity treatment (46–48).

The FDA has specifically approved setmelanotide (Imcivree) for the management of chronic weight in individuals aged six and older who have obesity due to rare genetic disorders (49). This restriction explains its low utilization and high cost. Imcivree is not designed for the management of general obesity, which severely limits the patient population it can treat, unlike other obesity medications that target a broader demographic (49).

The pricing of anti-obesity medications presents a unique scenario, driven by several factors. Historically, there has been a lack of effective and tolerable medications available in the market. This gap, combined with the high costs and potential complications associated with bariatric surgery, has created a significant demand for alternative treatments. With the increasing prevalence of obesity globally, the introduction of new medications such as Wegovy and tirzepatide marked a turning point. In the first 2 years following their release, these medications accounted for a dramatic increase in prescriptions, representing 88% of total prescriptions in 2023. This surge reflects not only the growing need for effective obesity treatments but also the opportunity for innovation in this therapeutic area. The convenience of weekly injections with Wegovy and tirzepatide, combined with the proven efficacy and tolerability, makes these two options more practical for clinical use. It’s also significant how quickly the use of these more recent agents has increased. In a recently published study (50), tirzepatide (Mounjaro) and Wegovy had monthly user growth rates of over 200 and 100%, respectively. Despite just having data for 7 months, Mounjaro’s adoption trajectory was especially high, most likely because of its dual incretin receptor activity on GIP and GLP-1, which helps patients lose more weight (50). As its data grows, Mounjaro’s potential to dominate the market is further highlighted by its distinctive technique of action (50). Additionally, the rising awareness of obesity as a public health issue and a risk factor for several diseases further emphasizes the critical role of these drugs in current treatment protocols. Moreover, the COVID-19 pandemic may reinforce the importance of managing obesity. The market demand and the healthcare system’s readiness to adopt such solutions highlight the significant potential for addressing obesity through pharmacological interventions.

The spending on specialty drugs, including those used to treat obesity, continues to rise, it is essential to explore policy measures that can mitigate these costs. Current proposals aimed at curbing drug price increases, such as adjusting the Medicaid rebate cap and increasing the minimum rebate for high-cost drugs, have the potential to significantly reduce federal spending over the next decade. In a recent study, Mylona et al. (51) conducted a statewide cross-sectional analysis and found that beneficiaries of Medicare and Medicaid were significantly more likely to be obese than those with commercial insurance, with odds ratios of 1.26 (95% CI: 1.20–1.32) and 1.27 (95% CI: 1.22–1.32), respectively. Further research into the cost-effectiveness of these drugs, particularly in comparison to other treatment options (such as lifestyle interventions or bariatric surgery), is necessary to fully understand their value within the Medicaid system.

This study’s findings are subject to several limitations due to the nature of the data obtained from the national Medicaid pharmacy database of CMS. One significant limitation is the absence of patient-specific data, which prevents an assessment of the effectiveness and tolerability of anti-obesity medications based on individual health outcomes. The lack of demographic information, such as age, gender, and comorbid conditions, restricts the ability to analyze how different population groups may have responded to the treatments. Additionally, our data does not include patient-specific prescription information, such as the number of treatment cycles patients received or the indications for which the prescriptions were given.

Tirzepatide was incorporated into our analysis because of its expanding usage and proven effectiveness in treating obesity; however, it should be noted that Zepbound was only approved in late 2023, thus only a quarter of the data was available for analysis. The completeness of our findings regarding tirzepatide’s usage patterns in the treatment of obesity may have been compromised by these limitations. Additionally, the data lack state-level granularity, which prevents us from analyzing geographic variation in drug utilization or linking trends to specific Medicaid policies across states. Future studies using richer datasets with demographic and policy-level variables are needed to assess potential disparities in access and outcomes among Medicaid subpopulations.

Moreover, although Medicaid covers a considerable portion of the U.S. population, the results of this study are limited to Medicaid beneficiaries, who predominantly represent low- and middle-income individuals. Therefore, the generalizability of these findings to other populations, such as those with private insurance or the uninsured, is restricted. Additionally, expenditures are reported in nominal dollars (unadjusted for inflation); this approach reflects the actual financial outlays experienced by Medicaid in each respective year and is consistent with methodologies used in similar pharmacoepidemiologic research. However, it should be acknowledged that part of the long-term increase reflects inflation over 24 years, although the majority of the growth is due to increased utilization of newer high-cost AOMs.

Lastly, the study does not account for external factors that could have influenced drug utilization patterns, such as marketing efforts by pharmaceutical companies. Notably, the COVID-19 pandemic may have contributed to an increased uptake of obesity treatments, as obesity became widely recognized as a risk factor for severe COVID-19 outcomes, along with other health complications. Furthermore, the introduction and proven efficacy and tolerability of newer medications, such as Wegovy and tirzepatide, have likely driven a significant rise in prescriptions, indicating the growing demand for effective obesity treatments. Despite these limitations, the study provides valuable insights into the pricing and utilization trends of anti-o.

## Conclusion

The findings of this study reveal a significant rise in Medicaid utilization and spending on anti-obesity medications from 1999 to 2023. The introduction of newer drugs, including Wegovy, Saxenda, and tirzepatide, contributed to this trend, with Wegovy and tirzepatide accounting for the majority of market share in 2023. Prescription numbers boosted by over 6,600% while spending soared due to rising drug prices and increased demand. These trends reflect the growing burden of obesity and the escalating costs of its treatment. Policymakers and healthcare providers must be aware of these dynamics to ensure fair access to effective therapies while addressing the financial challenges posed by the rising costs of these medications.

## Data Availability

Publicly available datasets were analyzed in this study. This data can be found at: Medicaid CMS.
