# Supplementary material for: Spending, utilization, and price trends for anti-obesity medications in U.S. Medicaid programs: an empirical analysis from 1999 to 2023
Source: Front Med (Lausanne). 2025 Jul 16;12:1537181. doi: 10.3389/fmed.2025.1537181 (PMC12307327; doi:10.3389/fmed.2025.1537181)

Appendix

Figure S1: Utilization trends of the AOMs in Medicaid, focusing specifically on the years 2013 to 2023.

Figure S2: Spending trends of the AOMs in Medicaid, focusing specifically on the years 2013 to 2023.

Figure S3: Joinpoint regression for tirzepatide AOM utlization, reimbursement and spending in CMS.


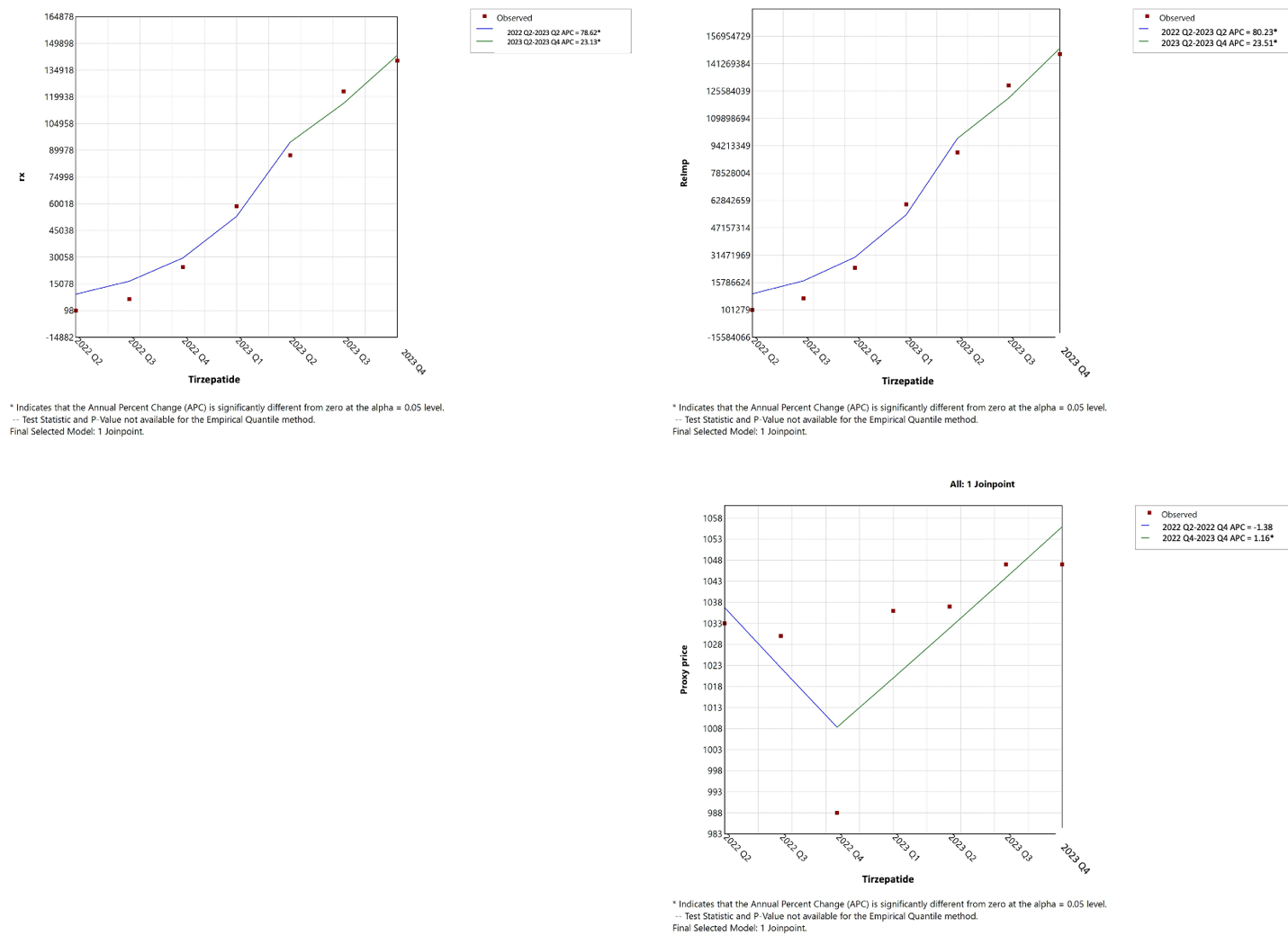


Figure S4: Joinpoint regression for Wegovy AOM utlization, reimbursement and spending in CMS.


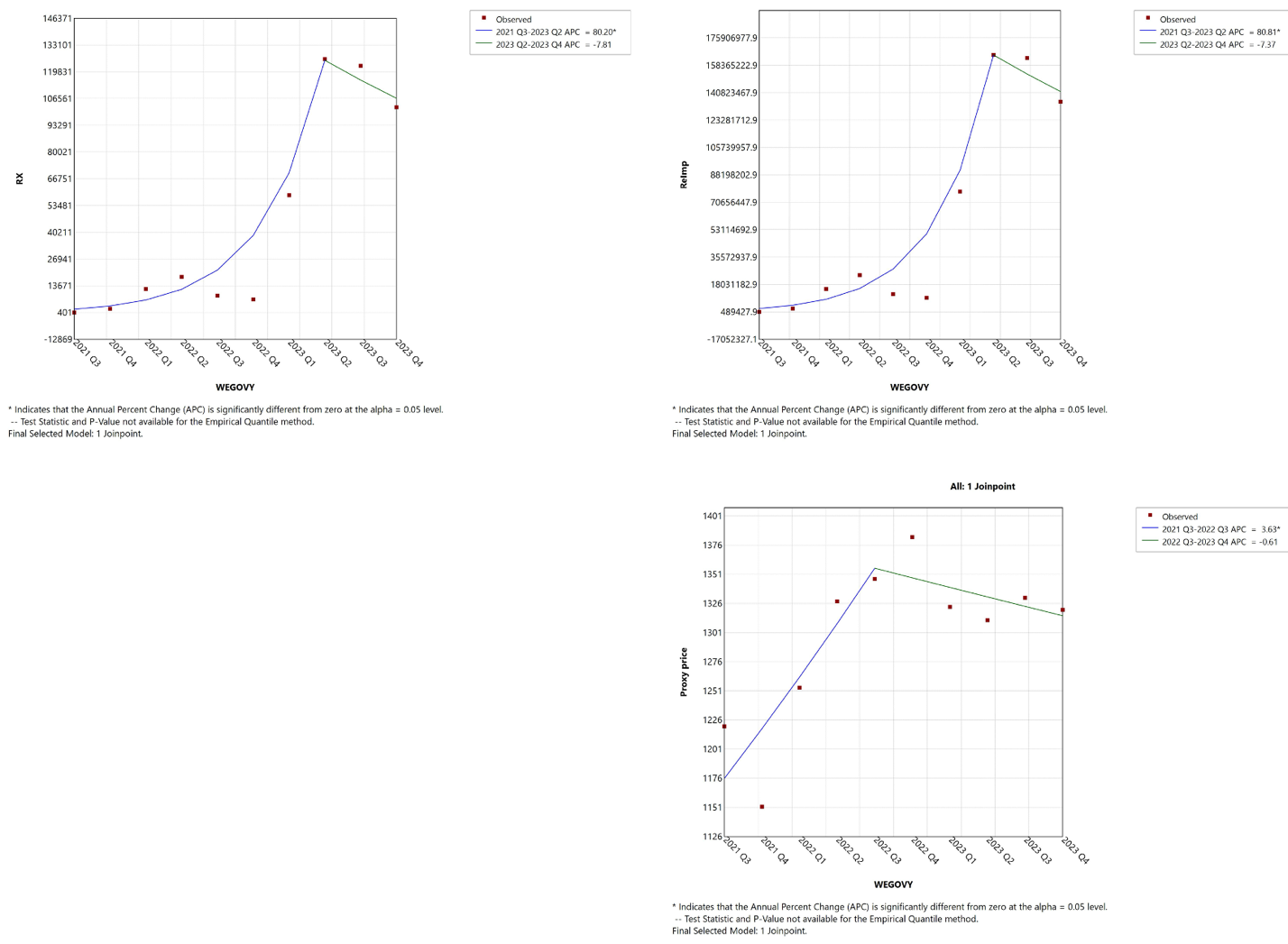


Figure S5: Joinpoint regression for Saxenda AOM utlization, reimbursement and spending in CMS.


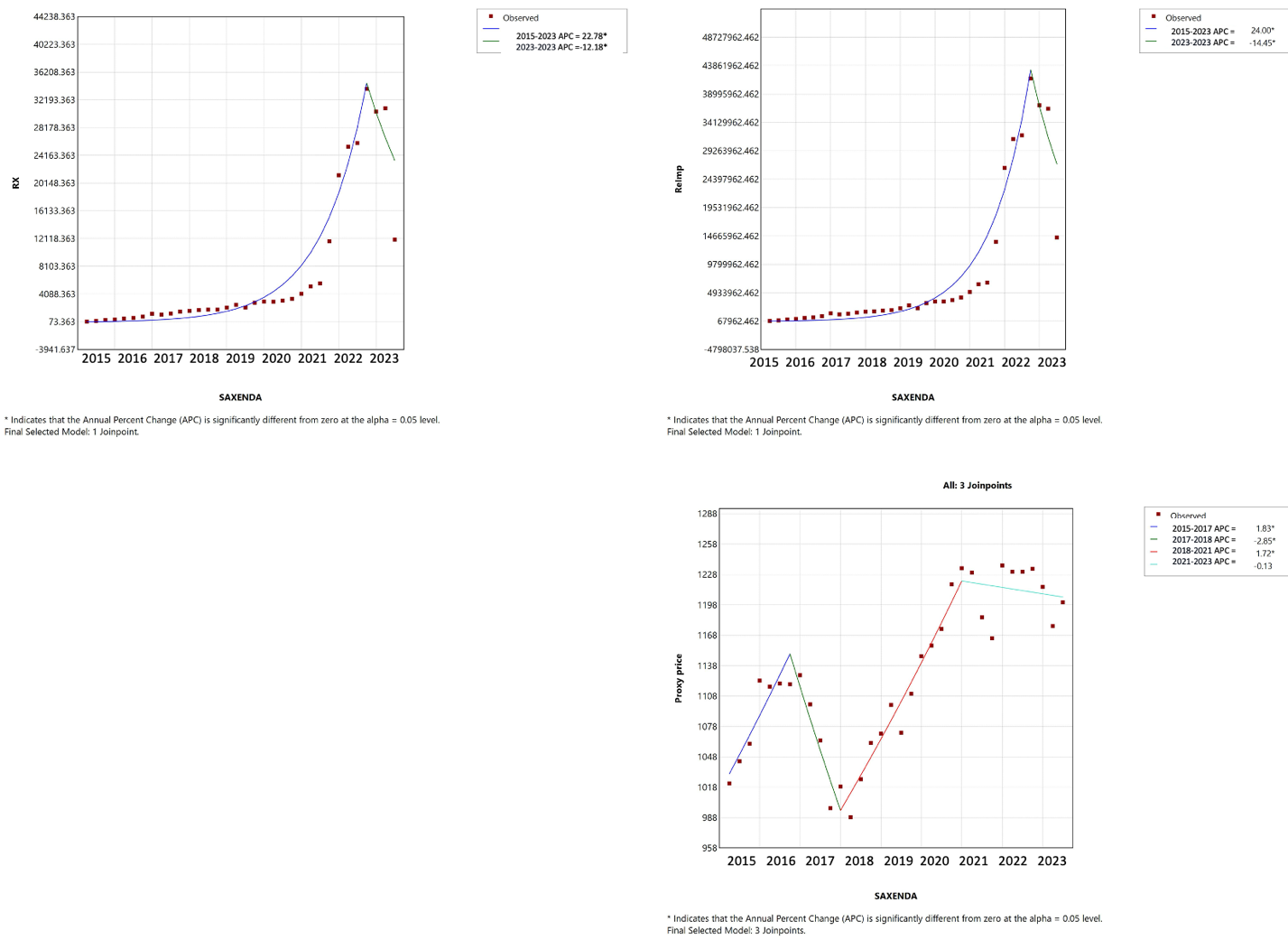


Figure S6: Joinpoint regression for Contrave AOM utlization, reimbursement and spending in CMS.


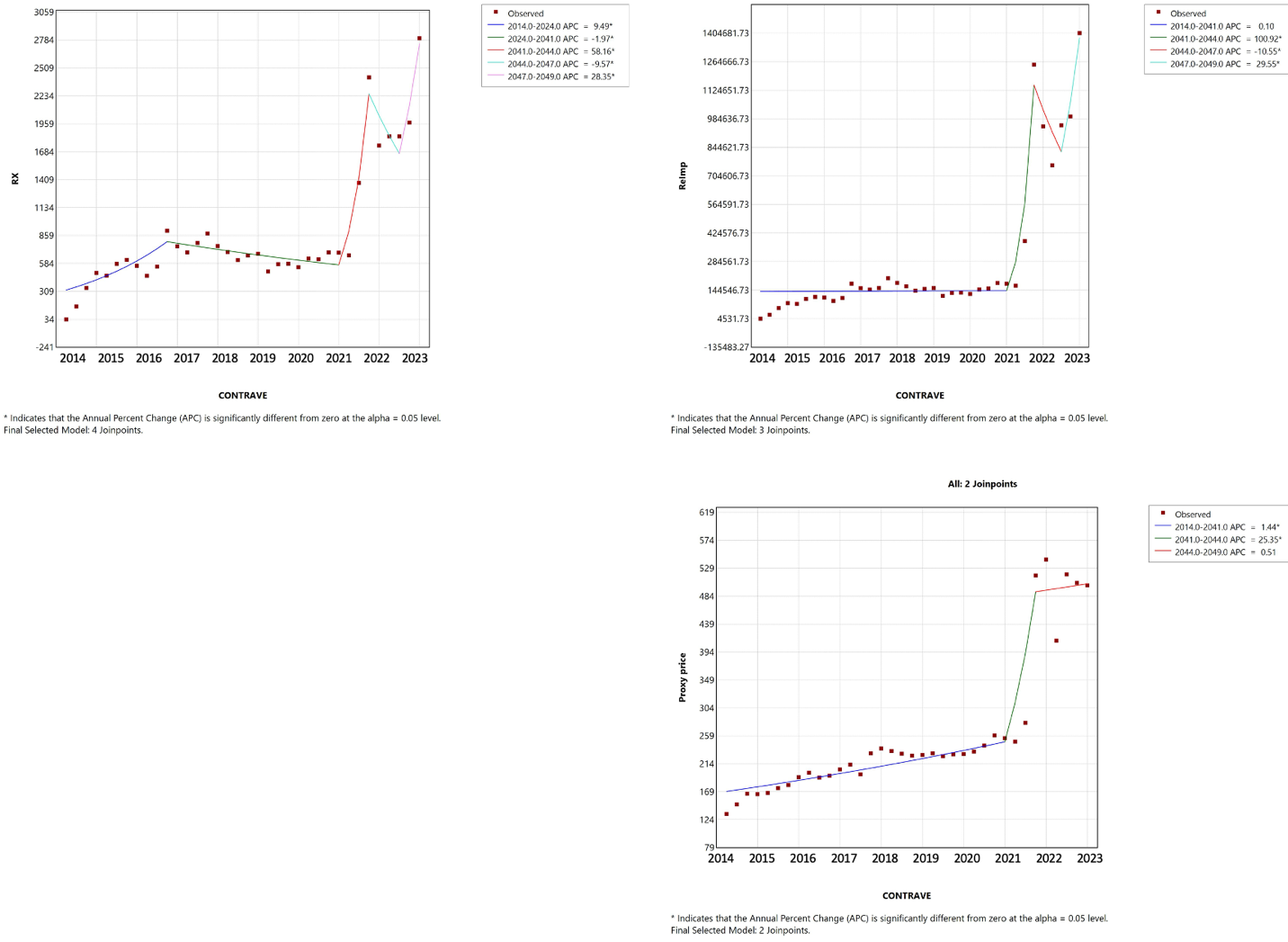


Figure S7: Joinpoint regression for Qsymia AOM utlization, reimbursement and spending in CMS.


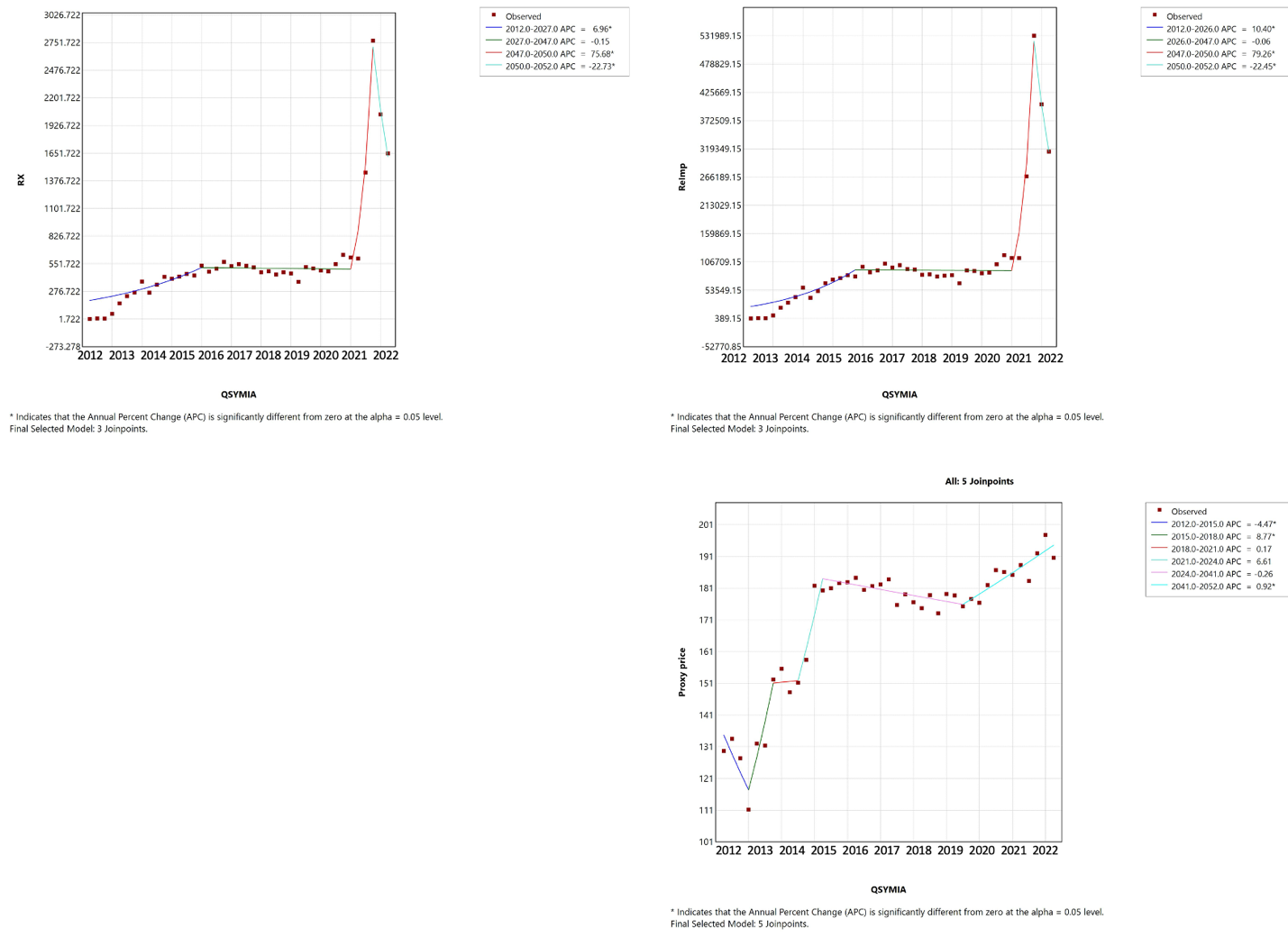


Figure S8: Joinpoint regression for Orlistat AOM utlization and spending in CMS.


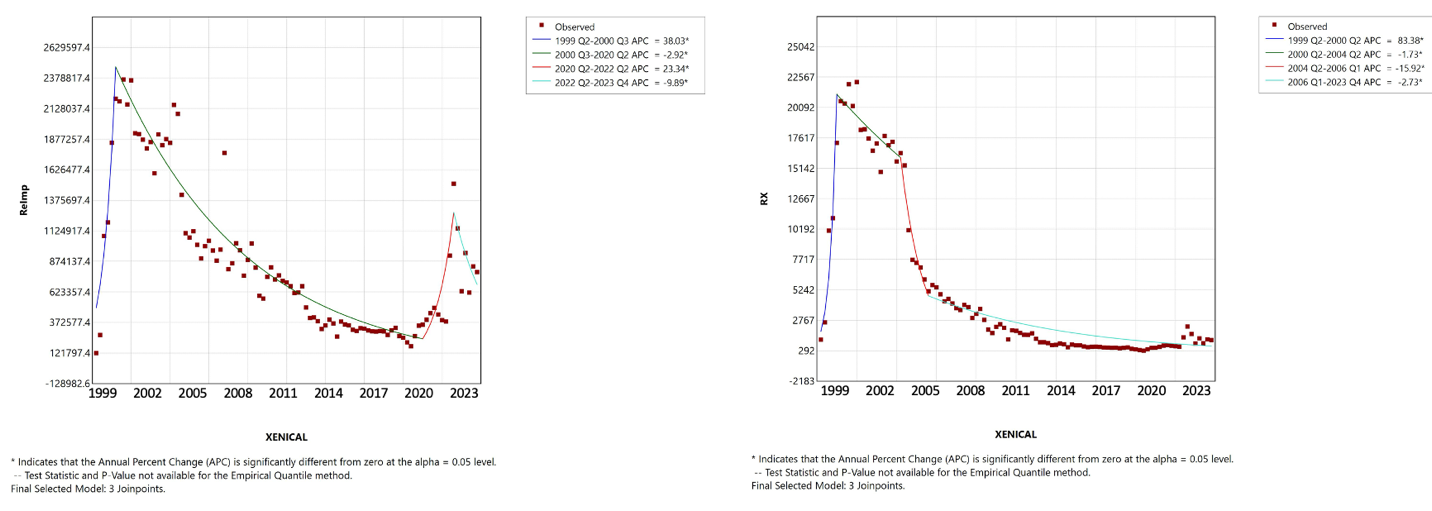


Figure S9: Joinpoint regression for Imcivree AOM utlization, reimbursement and spending in CMS.


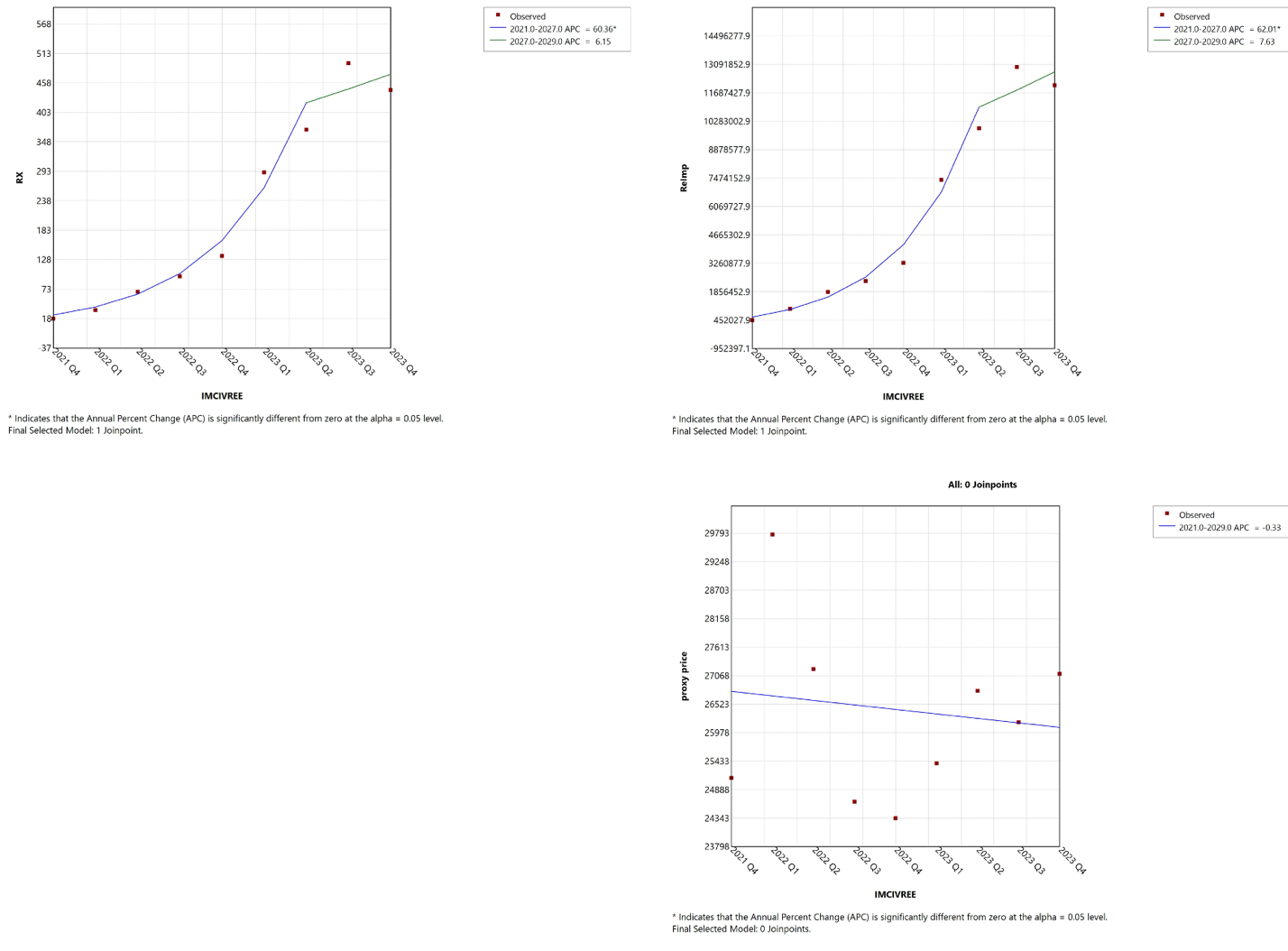


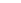

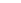

Supplement: Supplementary file 1 [file Data_Sheet_1.docx]
